# Bladder Dysfunction in an Obese Zucker Rat: The Role of TRPA1 Channels, Oxidative Stress, and Hydrogen Sulfide

**DOI:** 10.1155/2019/5641645

**Published:** 2019-08-20

**Authors:** Igor Blaha, María Elvira López-Oliva, María Pilar Martínez, Paz Recio, Ángel Agis-Torres, Ana Cristina Martínez, Sara Benedito, Albino García-Sacristán, Dolores Prieto, Vítor S. Fernandes, Medardo Hernández

**Affiliations:** ^1^Departamento de Urología, Hospital General Universitario Gregorio Marañón, 28007 Madrid, Spain; ^2^Departamento de Fisiología, Facultad de Farmacia, Universidad Complutense de Madrid, 28040 Madrid, Spain; ^3^Departamento de Anatomía y Embriología, Facultad de Veterinaria, Universidad Complutense de Madrid, 28040 Madrid, Spain

## Abstract

**Purpose:**

This study investigates whether functionality and/or expression changes of transient receptor potential vanilloid 1 (TRPV1) and transient receptor potential ankyrin 1 (TRPA1) channels, oxidative stress, and hydrogen sulfide (H_2_S) are involved in the bladder dysfunction from an insulin-resistant obese Zucker rat (OZR).

**Materials and Methods:**

Detrusor smooth muscle (DSM) samples from the OZR and their respective controls, a lean Zucker rat (LZR), were processed for immunohistochemistry for studying the expression of TRPA1 and TRPV1 and the H_2_S synthase cystathionine beta-synthase (CBS) and cysthathionine-*γ*-lyase (CSE). Isometric force recordings to assess the effects of TRPA1 agonists and antagonists on DSM contractility and measurement of oxidative stress and H_2_S production were also performed.

**Results:**

Neuronal TRPA1 expression was increased in the OZR bladder. Electrical field stimulation- (EFS-) elicited contraction was reduced in the OZR bladder. In both LZR and OZR, TRPA1 activation failed to modify DSM basal tension but enhanced EFS contraction; this response is inhibited by the TRPA1 blockade. In the OZR bladder, reactive oxygen species, malondialdehyde, and protein carbonyl contents were increased and antioxidant enzyme activities (superoxide dismutase, catalase, GR, and GPx) were diminished. CSE expression and CSE-generated H_2_S production were also reduced in the OZR. Both TRPV1 and CBS expressions were not changed in the OZR.

**Conclusions:**

These results suggest that an increased expression and functionality of TRPA1, an augmented oxidative stress, and a downregulation of the CSE/H_2_S pathway are involved in the impairment of nerve-evoked DSM contraction from the OZR.

## 1. Introduction

Transient receptor potential (TRP) channels are a membrane protein superfamily, mostly nonselective cation channels, present on primary sensory neurons that act as polymodal cellular sensors and are involved in somatosensory processes, such as the transduction of noxious mechanical, chemical, or thermal stimuli [1, 2]. In the lower urinary tract, transient receptor potential vanilloid 1 (TRPV1) and transient receptor potential ankyrin 1 (TRPA1) channels, present on capsaicin-sensitive primary afferents (CSPA), are involved in sensory bladder function and dysfunction, thus being considered as possible therapeutic targets in bladder disease [1, 2]. TRPV1 regulate the frequency of bladder reflex contractions, via direct stimulation of sensory fibers or through stretch-induced release of ATP from the urothelial cells [3, 4]. Under pathophysiological conditions, an increased TRPV1-dependent afferent nerve activity correlates with the development of an overactive bladder (OAB) [5]. Recently, a role for TRPV1 in urinary bladder dysfunction in a model of streptozotocin- (STZ-) induced diabetes has also been suggested. Thus, in the diabetic rat, an increased TRPV1 expression enhances micturition reflex arc functioning and a diminished TRPV1-dependent contraction contributes to bladder dysfunction [6]. TRPA1 activation elicits pain, protective reflexes, and local release of neurotransmitters in the periphery [7]. These receptors are expressed in urothelial cells and primary afferents of the bladder and urethra, and the increased expression of TRPA1 in the bladder wall is associated with the establishment of OAB and lower urinary tract symptoms (LUTS) [1, 2]. In the type 1 diabetic rat model induced by STZ, an enhanced TRPA1-dependent mechanism is involved in changes of detrusor smooth muscle (DSM) contractility as a consequence of the produced inflammatory reaction which leads to an increase in cyclooxygenase-2-dependent prostaglandin synthesis [8]. In addition to these TRP channels, diabetic micturition disorder is also associated with increased hyperglycemia-induced oxidative stress of the bladder [9] and reduced plasmatic H_2_S concentration [10].

Metabolic syndrome (MS) and type II diabetes mellitus (T2DM) are known risk factors for OAB and LUTS [11, 12]. An obese Zucker rat (OZR) is a genetic model of insulin resistance and MS caused by a dysfunctional gene of the leptin receptor [13]. T2DM, obesity, and complications arising therefrom alter the normal function of the lower urinary tract in the OZR, which makes them an excellent model for the study of bladder dysfunction associated with MS in men and women. In the OZR, the bladder wall is characterized by the presence of edema, vasculopathy, and an altered functionality of ion channels in DSM cells [14]. In fact, nitro-fatty acids produced from unsaturated fatty acids by oxidative inflammatory reactions and acidic conditions in the presence of nitric oxide (NO) or nitrites can activate TRP channels on CSPA nerve endings, leading to increased bladder contractility [15]. Moreover, a diminished function and expression of neuronal cannabinoid CB_1_ and CB_2_ receptors as well as a lower nerve fiber density are also involved in impaired excitatory neurotransmission of the OZR bladder [16].

Although obesity and diabetes are risk factors for urinary incontinence [17], the pathophysiological mechanisms responsible for bladder dysfunction in MS are not well known. The current study investigates whether changes in the function and/or expression levels of TRPV1 and TRPA1 channels, oxidative stress, and H_2_S are involved in the impaired nerve-mediated DSM contraction from the OZR.

## 2. Materials and Methods

### 2.1. Animal Model

Eight to 10-week-old male OZR (fa/fa, *n* = 20) (Crl: ZUC (Orl)-Lepr^fa^) and their controls, lean Zucker rats (LZRs) (fa/-, *n* = 20) were obtained from Charles River Laboratories (Barcelona, Spain) and kept on *ad libitum* chow and water until they reached 18-20 weeks old. The experiments were approved by the Animal Experimentation Ethics Committee of Complutense University and conformed to the European Union Guidelines (European Union Directive, 2010/63) for the Care and the Use of Laboratory Animals, and all the experimental protocols were approved by the Institutional Animal Care and Use Committee of Madrid Complutense University.

### 2.2. Isolation of Bladder Samples

Rats were killed by cervical dislocation, and the bladders were removed and immersed in a physiological saline solution (PSS) at 4°C. PSS contained (in mM) NaCl 119, KCl 4.6, MgCl_2_ 1.2, NaHCO_3_ 24.9, glucose 11, CaCl_2_ 1.5, KH_2_PO_4_ 1.2, and EDTA (ethylenediamine tetraacetic acid) 0.027. The adjacent connective and fatty tissues were removed, and longitudinal strips were obtained from the LZR and OZR DSM. The number of experiments had been specified prior to the start of the study. We obtained two strips from each OZR and LZR bladder. One preparation was used for isometric force recording, and the other one was for immunofluorescence and western blot assays or endogenous H_2_S measurement.

### 2.3. Plasma Measurements

Serum total cholesterol (TC) and TAG concentrations were assayed using enzymatic/colorimetric assay kits (Biolabo S.A. Materlab, Madrid, Spain). Serum glucose was measured using the glucose oxidase method (Spinreact S.A., Girona, Spain). Serum insulin concentration was determined using a mouse ELISA kit (Invitrogen, Madrid, Spain). The homoeostasis model assessment-estimated insulin resistance (HOMA-IR), a measure of the insulin resistance status, was calculated using the following formula: nonfasting blood glucose (mg/ml) × nonfasting insulin (ng/ml)/22.5. The greater the HOMA-IR value, the higher the level of insulin resistance is.

### 2.4. Immunohistochemistry

DSM strips from the OZR and LZR were fixed in 4% paraformaldehyde and embedded in paraffin. Sections were obtained and incubated overnight at 4°C using polyclonal primary antibodies against CSE (sc-135203, 1 : 50, Santa Cruz Biotechnology Inc., Heidelberg, Germany) and CBS (sc-67154, 1 : 50, Santa Cruz Biotechnology Inc., Heidelberg, Germany). Slides were then washed with PBS and allowed to incubate at room temperature for 30 min with the biotinylated secondary antibody goat anti-rabbit (IgG-B) (sc-2040, 1 : 500, Santa Cruz Biotechnology Inc., Heidelberg, Germany). After washing, the sections were incubated in HRP for 30 min and reveled with 3,3′-DAB (Sigma-Aldrich, Madrid, Spain). The sections were then counterstained by Harris's hematoxylin, dehydrated, and mounted.

### 2.5. Double-Labeling Immunofluorescence

DSM samples were fixed in 4% formaldehyde and cryoprotected in 30% sucrose. The bladders were frozen in OCT compound and then cut into 5 *μ*m transversal sections. The specimens were blocked in PB containing 5% bovine serum albumin, 10% normal goat serum, and 0.3% Triton X-100 for 2 h at room temperature. Next, the sections were allowed to incubate with the primary antibodies rabbit anti-TRPA1 (ab58844, 1 : 100, Abcam, Cambridge, UK) [18] or rabbit anti-TRPV1 (ab31895, 1 : 200, Abcam, Cambridge, UK) [19] and mouse anti-PGP 9.5 (ab8189, 1 : 100, Abcam, Cambridge, UK) for 48 h. After washing, the sections were incubated with secondary antibodies Alexa Fluor 488 goat anti-mouse (A-11029, 1 : 200, Invitrogen, Madrid, Spain) and Alexa Fluor 594 goat anti-rabbit (A-11037, 1 : 200, Invitrogen, Madrid, Spain). The cell nuclei were stained using DAPI (P36935, Molecular Probes, Eugene, USA). The intensity of fluorescence was evaluated using free software, ImageJ (USA).

### 2.6. Western Blotting

Protein extracts (50-80 *μ*g) were mixed and boiled with Laemmli buffer and resolved in 10% or 15% polyacrylamide gel. The proteins were then transferred to a polyvinylidene fluoride membrane (GE Healthcare, Spain). Membranes were incubated in blocking buffer containing 5% nonfat dry milk for 1 h at room temperature followed by the incubation with the following primary antibodies: anti-TRPV1 (1 : 2000), anti-TRPA1 (1 : 300), anti-CSE (1 : 200), anti-CBS (1 : 1000), anti-MnSOD (1 : 1000), anti-CuZnSOD (1 : 1000), anti-catalase (1 : 2000), anti-GR (1 : 1000), and anti-GPx (1 : 500) (Santa Cruz Biotechnology Inc., Heidelberg, Germany). Next, membranes were washed in 0.05% Tween-20 and then incubated with proper peroxidase-conjugated secondary antibodies for 1 h at room temperature. Membranes were then washed with PBS, and specific proteins were revealed by chemiluminescence (ECL-kit, GE Healthcare, Madrid, Spain). The blots obtained were scanned using an Image-Quant LAS500 system (GE Healthcare, Madrid, Spain), and the intensity of the bands for each protein was determined by densitometric analysis using Quantity One v4.62 (Bio-Rad, Madrid, Spain). The relative protein levels were normalized to the intensity of *β*-actin loading blots.

### 2.7. Tension Recordings

Urothelium-intact DSM strips (~4 mm long and ~2 mm wide) were connected to isometric force transducers and to a micrometer screw in a temperature-controlled (37°C) chamber (DMT-820MS, Aarhus, Denmark) containing 5 ml PSS aerated with 95% O_2_ and 5% CO_2_ to reach a pH = 7. The signal was recorded using data acquisition PowerLab hardware and LabChart v5.3 software (ADInstruments, Heidelberg, Germany). Strips were subjected to a tension of 1.2 g and allowed to equilibrate during 60 min. Samples were then incubated for 30 min with propranolol (10 *μ*M) and N^G^-nitro-L-arginine (L-NOARG, 100 *μ*M), to block, respectively, beta-adrenoceptor- and NO-mediated DSM relaxation. EFS was performed on strip basal tension by generating rectangular pulses (1 ms duration and 20 s trains) with 75 mA constant current output (Cibertec CS20 stimulator, Barcelona, Spain). For each strip, the first control curve was obtained by increasing EFS frequencies from 0.5 to 8 Hz at 4 min intervals. Then, the bath PSS was replaced every 15 min in a total period of 90 min and incubated with ASP 7663 [20], TRPA1 agonist, for 10 min, and the second curve was constructed. Strips were then washed out again and treated with HC 030031 [21], TRPA1 antagonist plus ASP 7663, for 30 min, and the third curve was performed.

### 2.8. Oxidative Stress and Antioxidant Capacity

Bladders from both OZR and LZR were homogenized in a buffer containing 1.15% (*w*/*v*) KCl, 5 mM EDTA, 0.2 mM PMSF, 0.2 mM DTT, and 25 mM phosphate buffer at pH 7.4. The homogenates were then centrifuged at 1500 g for 10 min at 4°C. The protein concentration was obtained using the Lowry method (kit, Bio-Rad, Madrid, Spain). The lipid peroxidation (LPO) was measured by using a kit (LPO-586, Oxis International Inc., N Camden, CA, USA). Carbonyl groups were measured by spectrophotometry, and reactive oxygen species (ROS) were measured by fluorometry using a kit (bioNova científica, Madrid, Spain).

The total activity of superoxide dismutase (SOD) was evaluated by the nitroblue tetrazolium method. Catalase (CAT) activity was measured at 240 nm using H_2_O_2_ as substrate. Selenium-dependent glutathione peroxidase (GPx) activities were determined and measured using the *tert*-butyl hydroperoxide subtraction. Glutathione reductase (GR) activity was assessed through the NADPH oxidation and measured at 340 nm. The measurement of the reduced glutathione (GSH) and glutathione disulfide (GSSG) was carried out using the commercial kit (Cayman Chemical, Michigan, USA).

### 2.9. Endogenous H_2_S Measurement

Tissues were homogenized (1 : 10, *w*/*v*) at 4°C in lysis buffer containing 50 mM of potassium phosphate at pH 6.8. Next, 50 mg of the bladder homogenate was placed in a 1 ml chamber containing the incubation solution (10 mM L-cysteine, 2 mM pyridoxal 5′-phosphate, and 100 mM potassium phosphate buffer at pH 7.4). DL-Propargylglycine (PPG, 1 mM) or aminooxyacetic acid (AOAA, 1 mM), inhibitors of cystathionine *γ*-lyase (CSE), and cystathionine *β*-synthase (CBS), respectively, were used to determine the role of these enzymes in the production of H_2_S. To initiate the reaction, the tubes containing the cocktail were placed in a shaking water bath at 37°C and allowed to incubate for 30 min. Next, 1% of zinc acetate was introduced to trap-generated H_2_S, and the reaction was stopped with trichloroacetic acid (500%, *w*/*v*). Then, N,N-dimethyl-p-phenylenediamine sulfate (20 mM; 0.5 ml) in 7.2 M HCl was added, followed by the subsequent addition of FeCl_3_ (30 mM; 0.4 ml) in 1.2 M HCl. The absorbance of 670 nm by the resulting solution was measured 20 min later by spectrophotometry. The total H_2_S concentration was determined from the standard curve obtained from NaHS and was expressed as nanomoles per mg of protein per 20 min. Protein was determined with the Lowry assay (Bio-Rad, Madrid, Spain).

### 2.10. Drugs and Solutions

The following drugs were used: aminooxyacetic acid (AOAA), DL-propargylglycine (PPG), N^G^-nitro-L-arginine (L-NOARG), and (±)-propranolol hydrochloride, and were purchased from Sigma (Madrid, Spain). (2E)-2-[7-Fluoro-1,2-dihydro-1-(2-methylpropyl)-2-oxo-3H-indol-3-ylidene]acetic acid (ASP 7663) and 2-(1,3-dimethyl-2,6-dioxo-1,2,3,6-tetrahydro-7H-purin-7-yl)-N-(4-isopropylphenyl)acetamide (HC 030031) were from Tocris (Madrid, Spain). The stock solutions of AOAA, ASP 7663, HC 030031, and PPG were prepared in dimethylsulfoxide (DMSO), and the final concentration of DMSO in the chambers did not exceed 1%. The rest of the compounds were dissolved in distilled water.

Physiological saline solution (PSS) composition was (mM) NaCl 119, KCl 4.6, MgCl_2_ 1.2, NaHCO_3_ 24.9, glucose 11, CaCl_2_ 1.5, KH_2_PO_4_ 1.2, and EDTA (ethylenediamine tetraacetic acid) 0.027. Potassium-rich PSS (KPSS) was PSS with KCl exchanged for NaCl on an equimolar basis.

### 2.11. Statistical Analysis

Data are presented as the mean ± SD of *n* rats. For the statistical differences between the OZR and LZR groups, we used unpaired two-sample (two-tailed) Student's *t*-test, whereas for multiple comparisons, one-way analysis of variance (ANOVA) followed by the Bonferroni post hoc test was applied. *P* values are descriptive. Statistical significances were assigned at *P* < 0.05.

## 3. Results

### 3.1. Body Weight, Biochemical Parameters, and Blood Pressure in OZR

At 18 weeks of age, OZR showed augmentation in body weight (359 ± 5 g and 491 ± 7 g in the LZR and OZR, respectively, *P* = 0.0001*vs.* the LZR value, unpaired Student's *t*-test, *n* = 20 from 20 LZR and OZR). Plasma blood glucose after nonfasting and insulin levels increased in the OZR (101 ± 2 mg/ml and 145 ± 28 mg/ml, *P* = 0.004*vs.* the control value, and 1.27 ± 0.34 ng/ml and 4.39 ± 0.95 ng/ml, *P* = 0.0001 vs. the control value, for glucose and insulin in the LZR and OZR, respectively, unpaired Student's *t*-test, *n* = 20 from 20 LZR and OZR). The HOMA-IR was significantly higher in the OZR group than the control group, indicating an increased insulin resistance in the OZR (5.2 ± 1.8 and 28.3 ± 8.2 in the LZR and OZR, respectively, *P* = 0.0003*vs.* the LZR value, unpaired Student's *t*-test, *n* = 20 from 20 LZR and OZR). Cholesterol and triglyceride plasma concentrations were also increased in the OZR (0.97 ± 0.11 mg/ml and 1.89 ± 0.21 mg/ml, *P* = 0.0001 vs. the control value, and 1.0 ± 0.2 mg/ml and 3.4 ± 1.3 mg/ml, *P* = 0.0005 vs. the control value, for cholesterol and triglycerides in the LZR and OZR, respectively, unpaired Student's *t*-test, *n* = 20 from 20 LZR and OZR). Therefore, OZR were consistently heavier than the LZR and exhibited mild hyperglycemia, hyperinsulinemia, and dyslipidemia with elevated total cholesterol and triglyceride levels. Systolic blood pressure was similar in the LZR and OZR (127 ± 3 mm and 131 ± 6 mm Hg, respectively, *n* = 20).

### 3.2. Expression of TRPV1 and TRPA1

**Figure 1:**
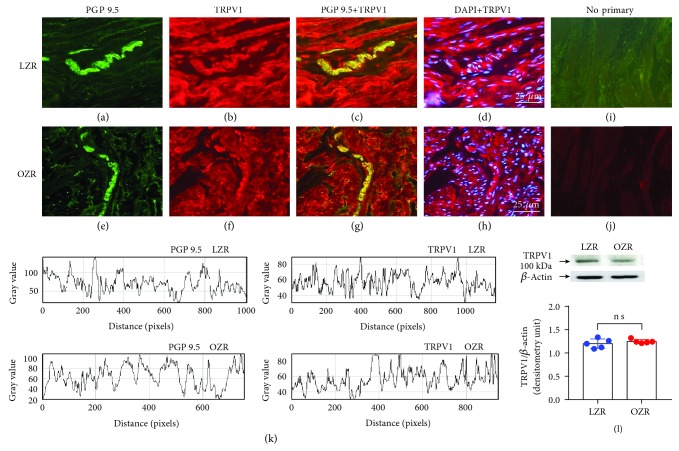
Similar TRPV1 expression in bladders from the LZR and OZR. TRPV1 expression in representative detrusor muscle transverse sections from a total of 5–6 lean (LZR) and obese (OZR) Zucker rat bladders. Detrusor overall innervation was visualized using the general nerve marker PGP 9.5 (green areas) (a and e). Bladder TRPV1 channel immunofluorescence from the LZR (b) and OZR (f) reveals immunopositive nerve trunks (red areas), running parallel to the smooth muscle bundles. Same fields (a, b, e, and f). Immunofluorescence double labelling for the PGP 9.5 and TRPV1 channel in the smooth muscle, demonstrating neuronal colocalization (yellow areas) (c and g). Cell nuclei were counterstained with DAPI (blue areas) (d and h). Scale bars indicate 25 *μ*m. Negative controls showing the lack of immunoreactivity in sections incubated in the absence of the primary antibody (i and j) (*n* = 5). Comparison of fluorescence density of nerve fibers and TRPV1 in the LZR and OZR, by using ImageJ, showing a similar expression of TRPV1 in nerve fibers of the LZR and OZR bladders (k). Western blot of detrusor smooth muscle membranes from the 5 LZR and OZR incubated with the TRPV1 antibody showing a 100 kDa major band, which corresponded to the expected molecular weight, suggesting there is no significant changes of TRPV1 protein expression in the LZR and OZR bladders (l). *P* = 0.0374*vs.* the LZR value, unpaired Student's *t*-test.

**Figure 2:**
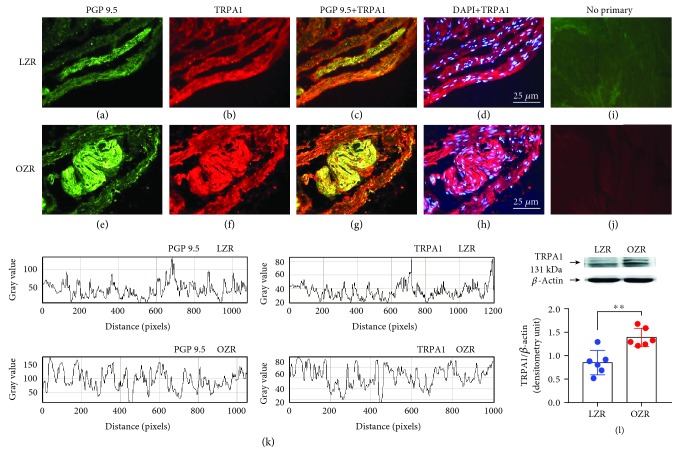
Increased TRPA1 expression in the OZR bladder. TRPA1 expression in representative detrusor muscle transverse sections from a total of 5–6 lean Zucker rat (LZR) and obese Zucker rat (OZR) bladders. Detrusor overall innervation was visualized using the general nerve marker PGP 9.5 (green areas) (a and e). Bladder TRPA1 channel immunofluorescence from the LZR (B) and OZR (f) reveals immunopositive nerve trunks (red areas), running parallel to the smooth muscle bundles. Same fields (a, b, e, and f). Immunofluorescence double labelling for the PGP 9.5 and TRPA1 channel in the smooth muscle, demonstrating neuronal colocalization (yellow areas) (c and g). Cell nuclei were counterstained with DAPI (blue areas) (d and h). Scale bars indicate 25 *μ*m. Negative controls showing the lack of immunoreactivity in sections incubated in the absence of the primary antibody (i and j) (*n* = 5). Comparison of the fluorescence density of nerve fibers and TRPA1 in the LZR and OZR, by using ImageJ, showing a higher expression of TRPA1 in nerve fibers of the OZR bladder (k). Western blot of detrusor smooth muscle membranes from the LZR and OZR incubated with the TRPA1 antibody showing a 131 kDa major band, which corresponded to the expected molecular weight, indicating an increased TRPA1 protein expression in the OZR bladder (l) (*n* = 5). ^∗∗^*P* = 0.002*vs.* the LZR value, unpaired Student's *t*-test.

Clearly positive staining for TRPV1 (Figures 1(a)–1(h)) and TRPA1 (Figures 2(a)–2(h)) antibodies was noted in LZR and OZR bladders located in nerve trunks and nerve fibers running parallel to the DSM bundles (*n* = 5). Similar expression of TRPV1 (Figure 1(k)) in nerve fibers of LZR and OZR bladders and a higher expression of TRPA1 (Figure 2(k)) in nerve fibers of the OZR bladder were observed. Western blot of DSM membranes from the LZR and OZR shows 100 kDa and 131 kDa major bands corresponding with the molecular weight for TRPV1 (Figure 1(l)) and TRPA1 (Figure 2(l)), respectively, indicating an increased TRPA1 protein level expression (a 41% increase versus the control value), in the OZR bladder (*n* = 6, *P* = 0.002 vs. the LZR value, Student's *t*-test), whereas there are no significant changes of TRPV1 protein expression in the OZR bladder.

### 3.3. Functional Studies

**Figure 3:**
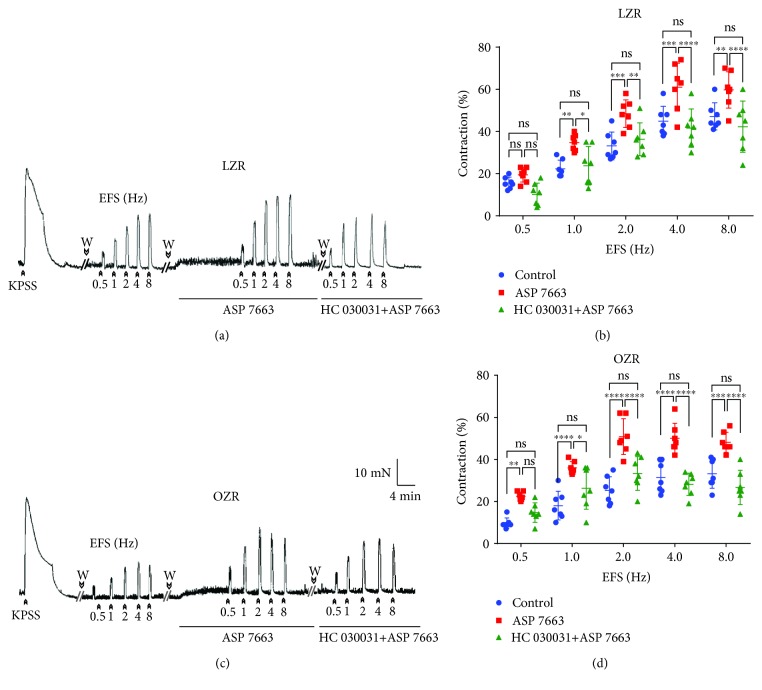
Increased TRPA1 activation on nerve-mediated contraction in the OZR bladder. Isometric force recordings showing the contractions evoked by potassium-rich (124 mM) physiological saline solution (KPSS) and electrical field stimulation (EFS, 1 ms in duration, 0.5 to 8 Hz, and 20-second trains), on the basal tone, of the lean Zucker rat (LZR) (a) and obese Zucker rat (OZR) (c) detrusor strips, treated with propranolol (10 *μ*M) and N^G^-nitro-L-arginine (100 *μ*M). Vertical bar shows tension in mN and horizontal bar time in min. W: wash. Frequency-response contraction curve to EFS in the detrusor from the LZR (open symbols) (b) and OZR (closed symbols) (d) in the absence (control, circles) or presence of ASP 7663 (10 *μ*M) (triangles), selective TRPA1 agonist, and ASP 7663 plus the TRPA1 antagonist, HC 030031 (60 *μ*M) (squares). Results are shown as mean ± SD percent of KPSS-induced contraction in 7 preparations from 7 rats. LZR at 1 Hz, ^∗∗^*P* = 0.007; 2 Hz, ^∗∗∗^*P* = 0.0007; 4 Hz, ^∗∗∗^*P* = 0.0003; 8 Hz, ^∗∗^*P* = 0.006; OZR at 1, 2, and 4 Hz, ^∗∗∗∗^*P* < 0.0001; 8 Hz, ^∗∗∗^*P* = 0.0001; ASP 7663 vs. control, *n* = 7. LZR at 1 Hz, ^∗^*P* = 0.002; 2 Hz, ^∗∗^*P* = 0.008; 4 Hz, ^∗∗∗∗^*P* = 0.0001; 8 Hz, ^∗∗∗∗^*P* = 0.0001; OZR at 1 Hz, ^∗^*P* = 0.019; 2, 4, and 8 Hz, ^∗∗∗∗^*P* = 0.0001; ASP 7663 vs. ASP 7663+HC 030031, *n* = 7; ASP 7663+HC 03003 vs. the control (ANOVA followed by the Bonferroni method).

DSM strips from both LZR (*n* = 7) and OZR (*n* = 7) were subjected to a passive tension of 9.8 ± 2.1 mN and allowed to equilibrate during 60 min. KPSS (124 mM)-induced contractions were similar in DSM from the LZR and OZR (37.3 ± 3.9 mN and 38.2 ± 2.9 mN in the LZR and OZR, respectively). ASP 7663 (10 *μ*M) [20] and HC 030031 (60 *μ*M) [22], TRPA1 agonist and antagonist, respectively, did not change the basal DSM tension from the LZR and OZR. EFS induced LZR and OZR bladder contractions in a frequency-dependent manner (0.5-8 Hz). The amplitude of these contractions was smaller in OZR DSM strips compared with those obtained in the LZR (contraction at 8 Hz of 47 ± 6.4% and 33 ± 6.9% in LZR and OZR bladders, respectively, *n* = 7, *P* = 0.0003*vs.* control, ANOVA) (Figure 3). In the LZR (Figures 3(a) and 3(b)) and OZR (Figures 3(c) and 3(d)), ASP 7663 increased the EFS responses and these effects were reversed by pretreatment of the DSM samples with ASP 7663 plus HC 030031. In the OZR, the potentiation of ASP 7663 on EFS contraction was higher with respect to that obtained in the LZR (25% and 44% increases of contraction at 8 Hz in the LZR and OZR, respectively, *n* = 7, *P* = 0.027*vs.* the LZR value, unpaired Student's *t*-test) (Figures 3(b) and 3(d)).

### 3.4. Oxidative Stress and Antioxidant Capacity in the OZR Bladder

**Figure 4:**
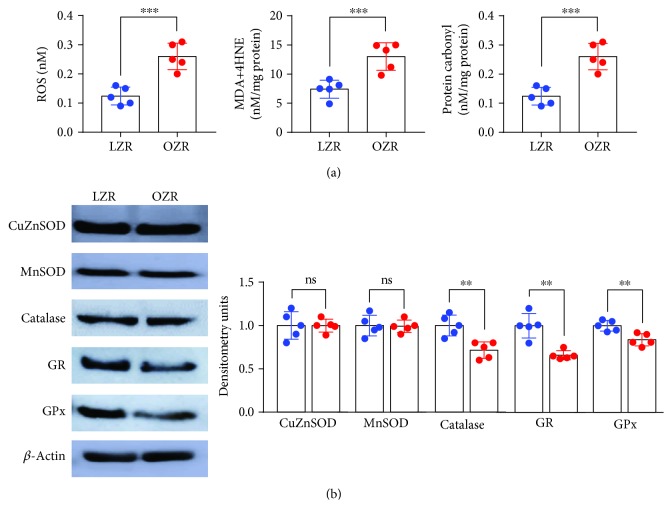
Augmented oxidative stress and diminished antioxidant enzyme activities in the OZR bladder. In the bladder from the OZR, the oxidative stress parameters, such as ROS production (141%), MDA (75%), and protein carbonyl content (109%), were significantly increased compared with those obtained in the LZR (a). In addition, the activities of the antioxidant enzymes SOD, catalase, GR, and GPx were diminished. Results are shown as mean ± SD from 5 preparations. ROS, ^∗∗∗^*P* = 0.0005*vs.* the LZR; MDA, ^∗∗^*P* = 0.002*vs.* the LZR; and protein carbonyl content, ^∗∗∗^*P* = 0.0005*vs.* the LZR value, unpaired Student's *t*-test (b).

In the bladder from the OZR, the oxidative stress parameters, such as ROS production, LPO level, and protein carbonyl content, were increased compared with those obtained in the LZR (Figure 4(a)). The total activity of SOD decreased in the OZR bladder, but the protein content of cytosolic CuZnSOD and mitochondrial MnSOD was not modified (Figure 4(b) and Table 1). In the OZR bladder, a decline was observed in both activities and the protein content (Figure 4(b)) of CAT, GPx, and GR and an increase (20%) of the SOD : CAT ratio compared with the control were observed (Table 1). In addition, GSH content in the OZR bladder decreased to 37%, whereas there were no changes in GSSG content between groups. Consequently, the GSH : GSSG ratio was decreased in the OZR bladder (Table 1).

### 3.5. CBS and CSE Expression and H_2_S Production

**Figure 5:**
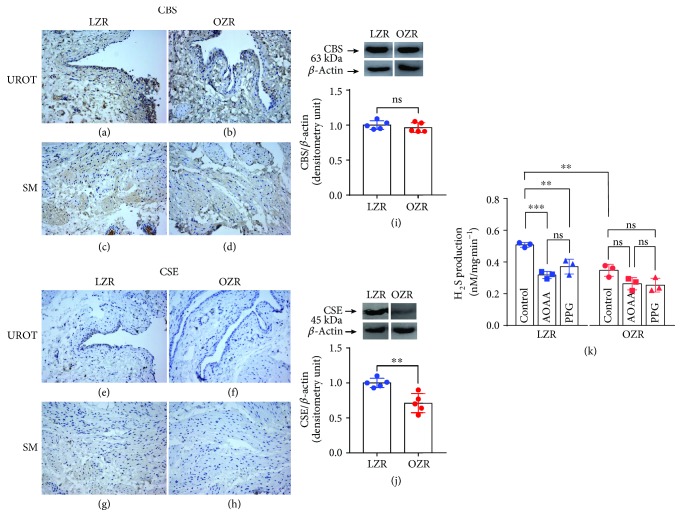
Downregulation of the CSE/H_2_S pathway in the OZR bladder. CBS and CSE expression in representative detrusor muscle transverse sections from a total of 5 lean Zucker rat (LZR) and obese Zucker rat (OZR) bladders (a–h). An intense CBS immunostaining in the smooth muscle (SM) and urothelium (URO) of bladders from the LZR and OZR was shown (a–d), whereas CSE staining was weaker (e–h). Both cytoplasm of the smooth muscle and urothelial cells of the bladder from the obese group (f and h) showed lower CSE staining versus that observed in the LZR (e and g). CBS immunoreactivity was similar in the LZR and OZR (a–d). Western blot of membranes from the LZR and OZR showing 63 kDa and 45 kDa major bands, which corresponded to the expected molecular weight for CBS and CSE, respectively, indicating a reduced CSE protein expression in the OZR bladder (j) (*n* = 5, ^∗∗^*P* = 0.003*vs.* the LZR value, unpaired Student's *t*-test). CBS protein expression was similar in bladders from the LZR and OZR (i). The level of H_2_S generated in the LZR and OZR bladders, in the absence (control) or presence of aminooxyacetic acid (AOAA, 1 mM) and DL-propargylglycine (PPG, 1 mM), inhibitors of CBS and CSE, respectively. Endogenous H_2_S production was significantly reduced in the OZR. Bars represent mean ± SD of the 3 LZR and OZR. ^∗^*P* = 0.003*vs.* LZR control values. ^∗∗∗^*P* = 0.0005 LZR control *vs.* LZR AOAA. ^∗∗^*P* = 0.008 LZR control *vs.* LZR PPG (ANOVA followed by the Bonferroni method) (k).

Both cytoplasm of the smooth muscle and urothelial cells of the bladder from the OZR (Figures 5(f) and 5(h)) showed lower CSE staining compared with that detected in the control group (Figures 5(e) and 5(g)). However, CBS immunoreactivity was similar in the LZR and OZR (Figures 5(a)–5(d)). Western blot of membranes from the LZR and OZR revealed two major bands at 63 kDa and 45 kDa, corresponding to the molecular weight for CBS and CSE, respectively, indicating diminished CSE protein expression levels in the OZR bladder (Figure 5(j)). There are no significant changes of CBS protein expression in bladders from the LZR and OZR (Figure 5(i)). In the OZR, endogenous H_2_S production was diminished compared with that obtained in the LZR. Blockade of CSE and CBS reduced the H_2_S level in the LZR but failed to modify the H_2_S generation in the OZR (Figure 5(k)).

## 4. Discussion

Current results suggest that increased functionality and expression of TRPA1, present at CSPA, augmented oxidative stress, diminished antioxidant enzyme activity, and CSE/H_2_S pathway downregulation are involved in the impaired nerve-evoked DSM contraction from the OZR. This conclusion is supported by the following findings. (i) In the OZR bladder, neuronal TRPA1 expression was consistently increased, whereas urothelial TRPA1 and pre- or postjunctional TRPV1 channel expression was similar to that found in the bladder from the LZR. (ii) Activation or blockade of TRPA1 failed to modify DSM basal tension from the LZR and OZR. (iii) EFS-induced contraction was consistently reduced in the bladder from the OZR versus that in the LZR. TRPA1 activation potentiates nerve-evoked DSM contraction from the LZR and OZR; this response is inhibited by TRPA1 blockade. Enhancement of EFS contraction produced by TRPA1 activation was higher in the OZR. (iv) In the OZR bladder, ROS formation, MDA, and protein carbonyl contents were increased and antioxidant enzyme activities were diminished. (v) The CSE-generated H_2_S level and CSE expression were reduced in the OZR bladder.

Vascular risk factors such as diabetes, obesity, atherosclerosis, dyslipidemia, hyperglycemia, and hypertension are related to the presence of LUTS [11]. In fact, obese persons show micturition disorders manifested by an increased presence of urge incontinence episodes caused by detrusor overactivity [17, 23]. In MS and T2DM, there are low levels of high-density lipoprotein, high triglyceride levels, atherosclerosis, and hypercholesterolemia with hypertension combined with hyperlipidemia, which are recognized risk factors for OAB [24]. Experimental animal models of high-fat diet-induced obesity show an increased expression of muscarinic M_2_ and M_3_ and purinergic P2X_3_ receptors in the urothelium and the smooth muscle, as well as the TRPV1 and inducible NO synthase proteins in the urothelium [12, 25]. All these protein expression changes have been related to the bladder mucosa oversensitivity and OAB in rats with MS [12, 25]. TRPV1 and TRPA1 have been identified in urothelial cells and CSPA of the bladder and urethra, which upon activation elicit pain, protective reflexes, and local release from afferents of inflammatory mediators, such as tachykinins and prostaglandins, which cause smooth muscle contraction [6, 8]. Increased expression of these cation channels in the bladder wall is associated with the development of OAB, cystitis-associated hypersensitivity, and LUTS [1, 2, 5]. In the current study, double-staining immunohistochemistry, using TRPV1 and TRPA1 channel selective antibodies combined with the neuronal marker PGP 9.5, and western blot assays showed a marked increase in the immunoreactivity and protein expression of TRPA1 located in nerve fibers distributed in the smooth muscle layer of the OZR bladder versus that observed in the LZR. No significant changes, however, in the pre- or postjunctional TRPV1 expression in the OZR bladder were observed. As neuronal receptor activation is involved in the release from CSPA of sensory contractile neuropeptides, such as tachykinins [8], the increased expression of prejunctional TRPA1 observed in the OZR bladder may lead to an enhanced nerve-evoked contraction of DSM from the OZR. However, in the genetic obesity model, purinergic and cholinergic neurogenic DSM contractions were consistently diminished according to the lower nerve fiber density observed, thus suggesting that both proinflammation and neuropathy of the OZR bladder, produced by metabolic perturbations, play a pivotal role in causing obesity-associated bladder dysfunction [16]. The diminished DSM nerve-mediated contraction obtained in the OZR is also consistent with increased bladder capacity, residual volume, and urethral resistance and decreased maximum detrusor contraction velocity and urine flow rate, considered to be detrusor underactivity-like symptoms responsible of bladder dysfunction in the diabetic fatty rat [26]. In the current study, urothelial TRPA1 expression in the OZR bladder was similar to that observed in the LZR. This together with the fact that activation and/or blockade of TRPA1 failed to modify consistently DSM tension from the LZR and OZR suggests that postjunctional TRPA1 seem not to play a significant role in impaired DSM contractility in the OZR.

In experimental models of diabetes mellitus, diabetic bladder dysfunction, as a common complication of diabetes, comprises both voiding symptoms, such as poor emptying and overflow incontinence, and storage symptoms, as urgency and urge incontinence which show a temporal effect on diabetic bladder dysfunction. Thus, early-phase diabetes mellitus produces compensated bladder function and the late phase causes decompensated bladder function. In models of T2DM, bladder dysfunction is essentially manifested as increased compliance; however, the relevance of bladder hypertrophy is questioned so that further investigations in such experimental models are needed [27].

Diabetic micturition disorder is frequently associated with increased hyperglycemia-induced oxidative stress of the bladder [9]. Oxidative stress is an excess of oxidant compounds caused by increased free radical production and/or decreased antioxidant defense systems that impairs cellular function and contributes to the late-stage failure of bladder function [9]. In fact, diabetes induced decrease in DSM force associated with increased oxidative stress and overactivity of aldose reductase. The oxidative damage of the smooth muscle cells results in protein damage, and the activation of apoptotic pathways contributes to diabetic bladder dysfunction. The substantial oxidative stress generated in diabetic rats, produced as a consequence of hyperglycemia and hyperlipidemia, correlates with increased urinary frequency and decreased bladder blood vessel and nerve density [9, 23, 24]. An increased oxidative stress and bladder ischemia cause bladder nerve dysfunction, nerve fiber injury, mitochondrial injury, and detrusor muscle cell damage [28]. Insulin resistance has been associated with increased oxidative stress-associated cellular damage [29]. In the present study, insulin resistance due to hyperinsulinemia and hyperglycemia in the OZR, as indicated by the increase in HOMA-IR, was paralleled with increased oxidative stress in the OZR bladder. Thus, ROS production and MDA+4-HNE and protein carbonyl contents, a marker of lipid peroxidation and an early and sensitive indicator of oxidative damage to proteins, respectively, were consistently augmented demonstrating the prooxidant potential of the OZR bladder. In addition, a decrease in the levels and activities of the antioxidant enzymes, such as SOD, catalase, GR, and GPx, was detected in the cytosolic fraction of the bladder wall tissue from the OZR. Overproduction of ROS in obesity may directly deplete antioxidant molecules, leading to apparently insufficient antioxidant capacity to compensate for oxidative stress. Antioxidant enzymes protect tissue against injury, and tissue damage occurs after the antioxidants are depleted. SOD represents the first barrier against the increase of superoxide anion formation in the mitochondria since that competes with NO for reaction with superoxide anions and prevents the generation of peroxinitrites, a potent oxidant that can modify proteins to form 3-nitrotyrosine [30]. Therefore, the inactivation of SOD could lead to self-amplification of oxidative stress in the bladder wall from the OZR progressively enhancing peroxinitrite production and secondary tissue damage, which may be involved in the diminished nerve-evoked DSM contraction from the OZR. The reduction in SOD activity, coupled with a decrease of catalase and GPx activities, indicates a functional fall in the capacity of dismutation of O_2_^−^ to H_2_O_2_ inducing the increase of free radicals. The decrease in both GR and GPx proteins and activities produced a decrease in the cytosolic GSH : GSSG ratio, altering the GSH redox status in the OZR bladder. These results agree with those described in the STZ-induced diabetic rat where a higher lipid peroxidation production as well as a decreased antioxidant enzyme activity supported the higher oxidative stress detected [31]. In the current study, the activation of TRPA1 in the OZR bladder promotes a higher increase of the nerve-mediated contraction in comparison with that obtained in the LZR. The potentiation produced by TRPA1 activation on impaired EFS contraction in the OZR bladder suggests that, in addition to increased TRPA1 receptor expression, an augmented functionality of TRPA1 receptors, located at CSPA, in the impaired excitatory neurotransmission from the OZR bladder seems to be involved in obesity-related micturition disorder.

High-fat diet, obesity, and insulin resistance modify the CBS/CSE/H_2_S system in the liver and adipose tissue. In fact, most studies indicate that plasmatic H_2_S concentration decreases in animal models of diabetes and in diabetic humans [10]. In the lower urinary tract, the action of endogenous H_2_S on sensory neurons has been widely documented. Thus, H_2_S generated by CSE is involved in the inhibitory neurotransmission of the pig bladder neck producing smooth muscle relaxation through the release of inhibitory neuropeptides, as pituitary adenylate cyclase-activating polypeptide 38 and/or calcitonin gene-related peptide from CSPA via TRPA1 and TRPV1 channel activation [22, 32]. In detrusor, H_2_S stimulates the release from sensory nerve endings of tachykinins to cause DSM contraction [33]. H_2_S is also an endogenous TRPA1 ligand involved in inflammatory bladder disease producing bladder pain and referred hyperalgesia via Cav3.2 T-type Ca^2+^ channel activation [34]. In the OZR bladder, endogenous H_2_S production and CSE expression were consistently reduced compared with those observed in the LZR, thus suggesting that H_2_S, generated by a downregulated CSE activity, may contribute to the obesity-associated bladder dysfunction.

In conclusion, present results suggest that increased expression and functionality of TRPA1, located at CSPA, excessive oxidative stress, and downregulation of the CSE/H_2_S pathway are involved in the impairment of nerve-evoked DSM contraction in the insulin-resistant OZR. Insulin resistance was likely a pivotal factor that contributes to the bladder dysfunction in obesity.

## Figures and Tables

**Table 1:** Levels of reduced glutathione (GSH), glutathione disulfide (GSSG), and the GSH : GSSG ratio; superoxide dismutase (SOD), catalase (CAT), glutathione reductase (GR), and glutathione peroxidase (GPx) activities; and both SOD : CAT and SOD : GPx ratios in the bladder from the lean Zucker rat (LZR) and obese Zucker rat (OZR).

	LZR	OZR	*P* value
GSH (nm/mg protein)	8.0 ± 1.0	5.0±1.37^∗∗^	0.004
GSSG (nm/mg protein)	0.08 ± 0.01	0.10 ± 0.01^∗^	0.043
GSH : GSSG	107 ± 13.37	48.7±9.03^∗∗∗∗^	<0.0001
SOD (U/min/mg protein)	18.2 ± 2.5	12.6±2.19^∗∗^	0.0051
CAT (U/min/mg protein)	12.6 ± 1.75	7.0±1.53^∗∗∗^	0.0007
GR (U/min/mg protein)	24.6 ± 2.64	20.5 ± 2.39^∗^	0.0317
GPx (U/min/mg protein)	16.6 ± 2.32	11.0±1.20^∗∗^	0.0013
SOD : CAT ratio	1.4 ± 0.23	1.8 ± 0.20^∗^	0.0290
SOD : GPx ratio	1.1 ± 0.03	1.1 ± 0.11	0.4088

Results represent the mean ± SD of 6 rats. Unpaired Student's *t*-test.

## Data Availability

The data used to support the findings of this study are included within the supplementary information file.

## Abbreviations

CBS: Cystathionine beta-synthase
CSE: Cysthathionine-*γ*-lyase
CSPA: Capsaicin-sensitive primary afferents
T2DM: Type II diabetes mellitus
DSM: Detrusor smooth muscle
EFS: Electrical field stimulation
H_2_S: Hydrogen sulfide
TRPA1: Transient receptor potential ankyrin 1
TRPV1: Transient receptor potential vanilloid 1
LUTS: Lower urinary tract symptoms
LZR: Lean Zucker rat
MS: Metabolic syndrome
OAB: Overactive bladder
OZR: Obese Zucker rat.

## Supplementary Materials

Supplementary 1 Supplementary Figure 1: similar TRPV1 expression in bladders from the LZR and OZR. Uncropped images of immunoblots of TRPV1 and *β*-actin displayed in Figure 1(l) in the lean Zucker rat (LZR) and obese Zucker rat (OZR) (*n* = 5 − 6). The bands of interest are indicated by black boxes on the gels and show a similar TRPV1 expression in bladders from the LZR and OZR.

Supplementary 2 Supplementary Figure 2: increased TRPA1 expression in the OZR bladder. Uncropped images of immunoblots of TRPA1 and *β*-actin displayed in Figure 2(l) in the lean Zucker rat (LZR) and obese Zucker rat (OZR) (*n* = 5‐6). The bands of interest are indicated by black boxes on the gels and show an increased TRPA1 expression in the OZR bladder.

Supplementary 3 Supplementary Figure 3: similar CuZnSOD and MnSOD expression in bladders from the LZR and OZR and a reduced catalase, GR, and GPx expression in the OZR bladder. Uncropped images of immunoblots of CuZnSOD, MnSOD, catalase, GR, GPx, and *β*-actin displayed in Figure 4(b) in the lean Zucker rat (LZR) and obese Zucker rat (OZR) (*n* = 6). The bands of interest are indicated by black boxes on the gels and show a similar CuZnSOD and MnSOD expression in bladders from the LZR and OZR and a reduced catalase, GR, and GPx expression in the OZR bladder.

Supplementary 4 Supplementary Figure 4: similar CBS expression in bladders from the LZR and OZR. Uncropped images of immunoblots of CBS and *β*-actin displayed in Figure 5(i) in the lean Zucker rat (LZR) and obese Zucker rat (OZR) (*n* = 6). The bands of interest are indicated by black boxes on the gels and show a similar CBS expression in bladders from the LZR and OZR.

Supplementary 5 Supplementary Figure 5: reduced CSE expression in the OZR bladder. Uncropped images of immunoblots of CSE and *β*-actin displayed in Figure 5(j) in the lean Zucker rat (LZR) and obese Zucker rat (OZR) (*n* = 6). The bands of interest are indicated by black boxes on the gels and show a reduced CSE expression in the OZR bladder.
