# Supplementary material for: Bladder Dysfunction in an Obese Zucker Rat: The Role of TRPA1 Channels, Oxidative Stress, and Hydrogen Sulfide
Source: Oxid Med Cell Longev. 2019 Aug 20;2019:5641645. doi: 10.1155/2019/5641645 (PMC6721245; doi:10.1155/2019/5641645)
Supplement: Supplementary 1 — Supplementary Figure 1: similar TRPV1 expression in bladders from the LZR and OZR. Uncropped images of immunoblots of TRPV1 and β-actin displayed in Figure 1(l) in the lean Zucker rat (LZR) and obese Zucker rat (OZR) (n = 5 − 6). The bands of interest are indicated by black boxes on the gels and show a similar TRPV1 expression in bladders from the LZR and OZR. [file 5641645.f1.pptx]

## Slide 1
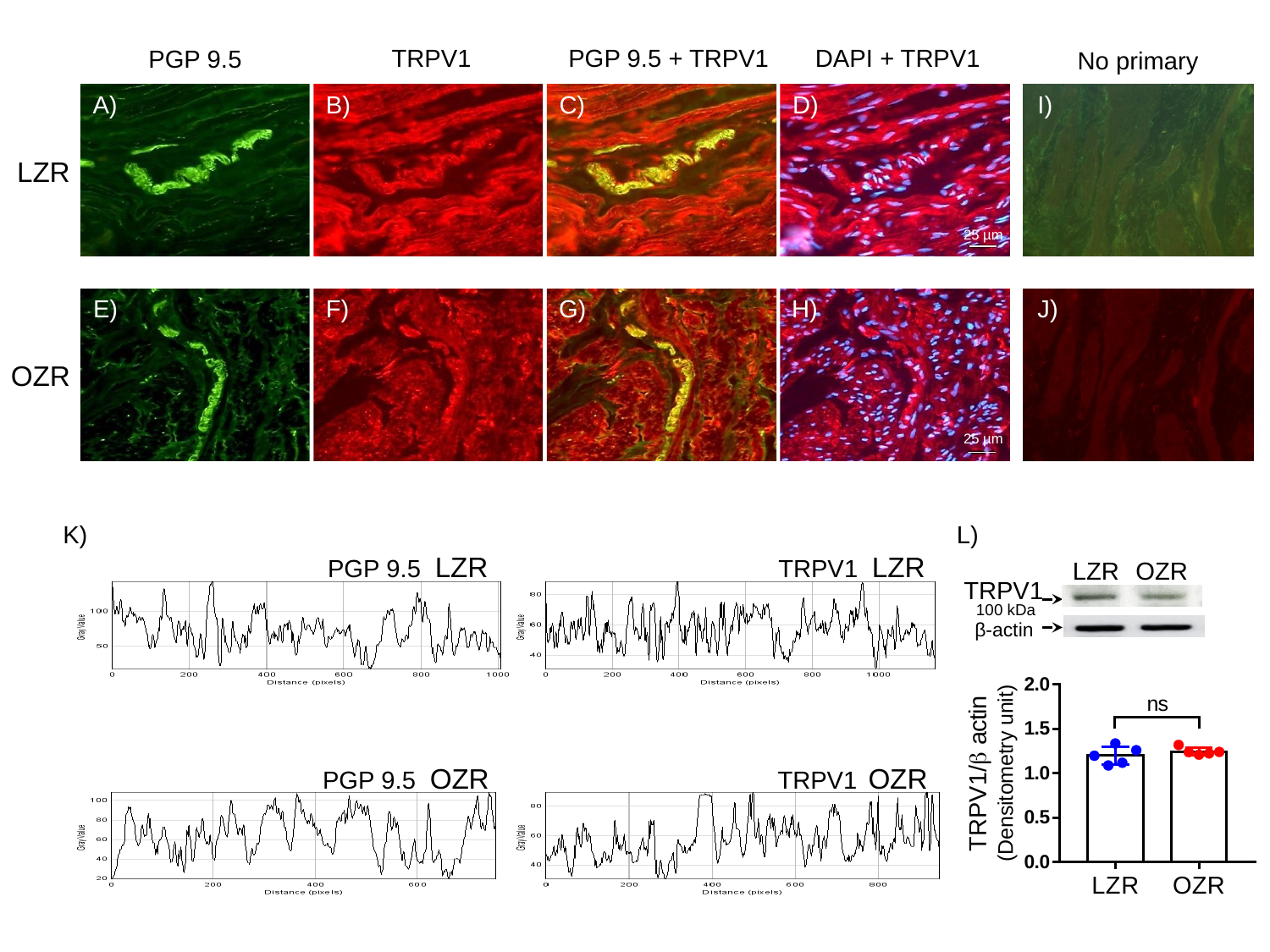

TRPV1
PGP 9.5 + TRPV1
DAPI + TRPV1
PGP 9.5
No primary
A)
B)
C)
D)
I)
LZR
25 µm
E)
F)
G)
H)
J)
OZR
25 µm
L)
K)
TRPV1 LZR
PGP 9.5 LZR
TRPV1 OZR
PGP 9.5 OZR
LZR
OZR
TRPV1
100 kDa
β-actin
LZR OZR
TRPV1
β-actin
