# Supplementary material for: Bladder Dysfunction in an Obese Zucker Rat: The Role of TRPA1 Channels, Oxidative Stress, and Hydrogen Sulfide
Source: Oxid Med Cell Longev. 2019 Aug 20;2019:5641645. doi: 10.1155/2019/5641645 (PMC6721245; doi:10.1155/2019/5641645)
Supplement: Supplementary 2 — Supplementary Figure 2: increased TRPA1 expression in the OZR bladder. Uncropped images of immunoblots of TRPA1 and β-actin displayed in Figure 2(l) in the lean Zucker rat (LZR) and obese Zucker rat (OZR) (n = 5‐6). The bands of interest are indicated by black boxes on the gels and show an increased TRPA1 expression in the OZR bladder. [file 5641645.f2.pptx]

## Slide 1
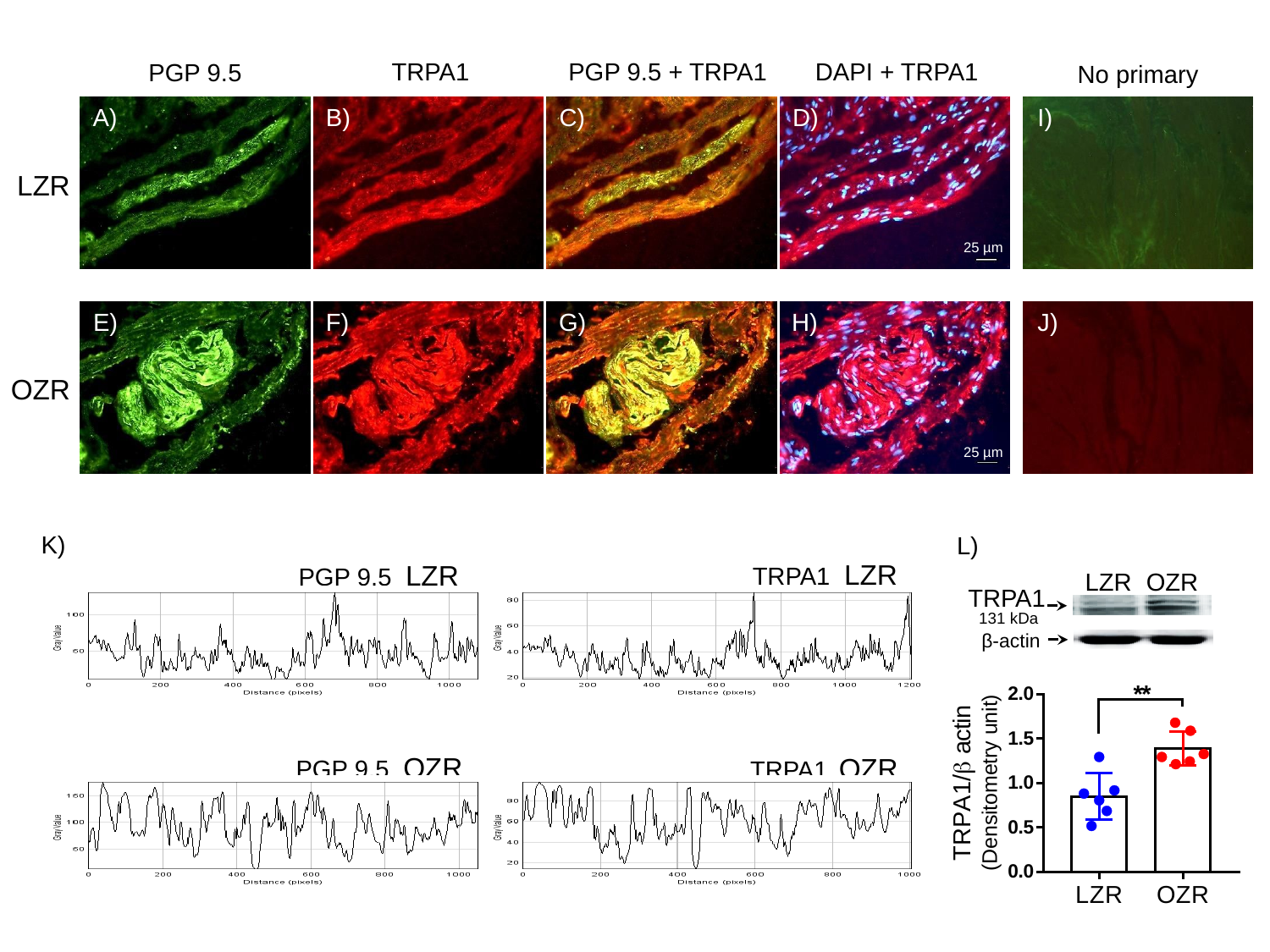

TRPA1
PGP 9.5 + TRPA1
DAPI + TRPA1
PGP 9.5
No primary
A)
B)
C)
D)
I)
25 µm
LZR
E)
F)
G)
H)
J)
25 µm
OZR
K)
TRPA1 LZR
PGP 9.5 LZR LZR
TRPA1 OZR
PGP 9.5 OZR OZR
L)
LZR
OZR
TRPA1
131 kDa
β-actin
LZR OZR
TRPA1
β-actin
