# Supplementary figures and images for: Bladder Dysfunction in an Obese Zucker Rat: The Role of TRPA1 Channels, Oxidative Stress, and Hydrogen Sulfide
Source: Oxid Med Cell Longev. 2019 Aug 20;2019:5641645. doi: 10.1155/2019/5641645 (PMC6721245; doi:10.1155/2019/5641645)

## Slide 1
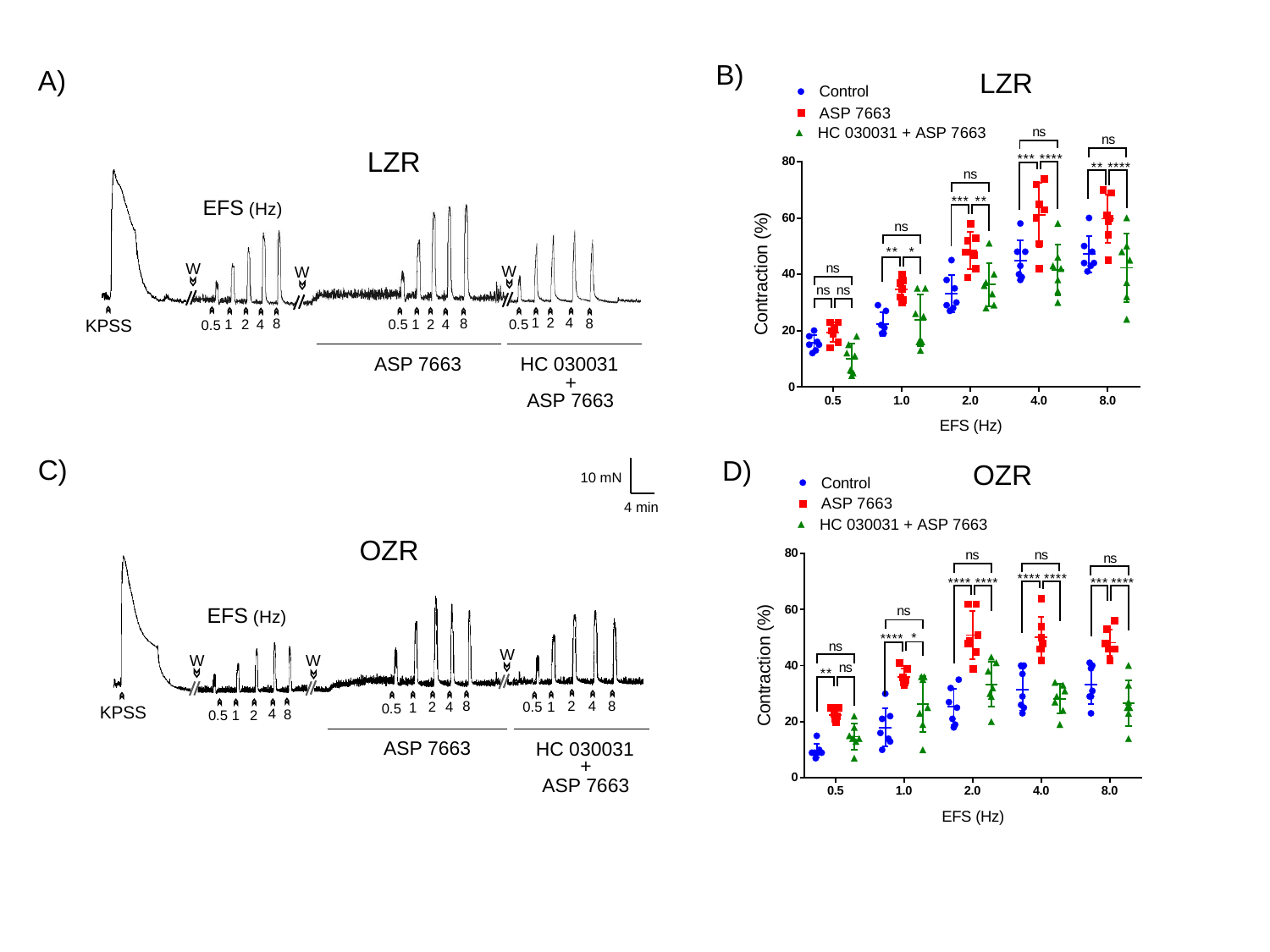

B)
A)
LZR
LZR
EFS (Hz)
W
W
W
2
1
4
KPSS
8
8
8
1
2
0.5
4
0.5
1
2
4
0.5
ASP 7663
HC 030031
+
ASP 7663
C)
D)
OZR
10 mN
4 min
OZR
EFS (Hz)
W
W
W
2
4
8
8
2
0.5
1
4
1
0.5
KPSS
4
8
2
0.5
1
ASP 7663
HC 030031
+
ASP 7663

Supplement: Supplementary 3 — Supplementary Figure 3: similar CuZnSOD and MnSOD expression in bladders from the LZR and OZR and a reduced catalase, GR, and GPx expression in the OZR bladder. Uncropped images of immunoblots of CuZnSOD, MnSOD, catalase, GR, GPx, and β-actin displayed in Figure 4(b) in the lean Zucker rat (LZR) and obese Zucker rat (OZR) (n = 6). The bands of interest are indicated by black boxes on the gels and show a similar CuZnSOD and MnSOD expression in bladders from the LZR and OZR and a reduced catalase, GR, and GPx expression in the OZR bladder. [file 5641645.f3.pptx]
