# Supplementary material for: Bladder Dysfunction in an Obese Zucker Rat: The Role of TRPA1 Channels, Oxidative Stress, and Hydrogen Sulfide
Source: Oxid Med Cell Longev. 2019 Aug 20;2019:5641645. doi: 10.1155/2019/5641645 (PMC6721245; doi:10.1155/2019/5641645)
Supplement: Supplementary 4 — Supplementary Figure 4: similar CBS expression in bladders from the LZR and OZR. Uncropped images of immunoblots of CBS and β-actin displayed in Figure 5(i) in the lean Zucker rat (LZR) and obese Zucker rat (OZR) (n = 6). The bands of interest are indicated by black boxes on the gels and show a similar CBS expression in bladders from the LZR and OZR. [file 5641645.f4.pptx]

## Slide 1
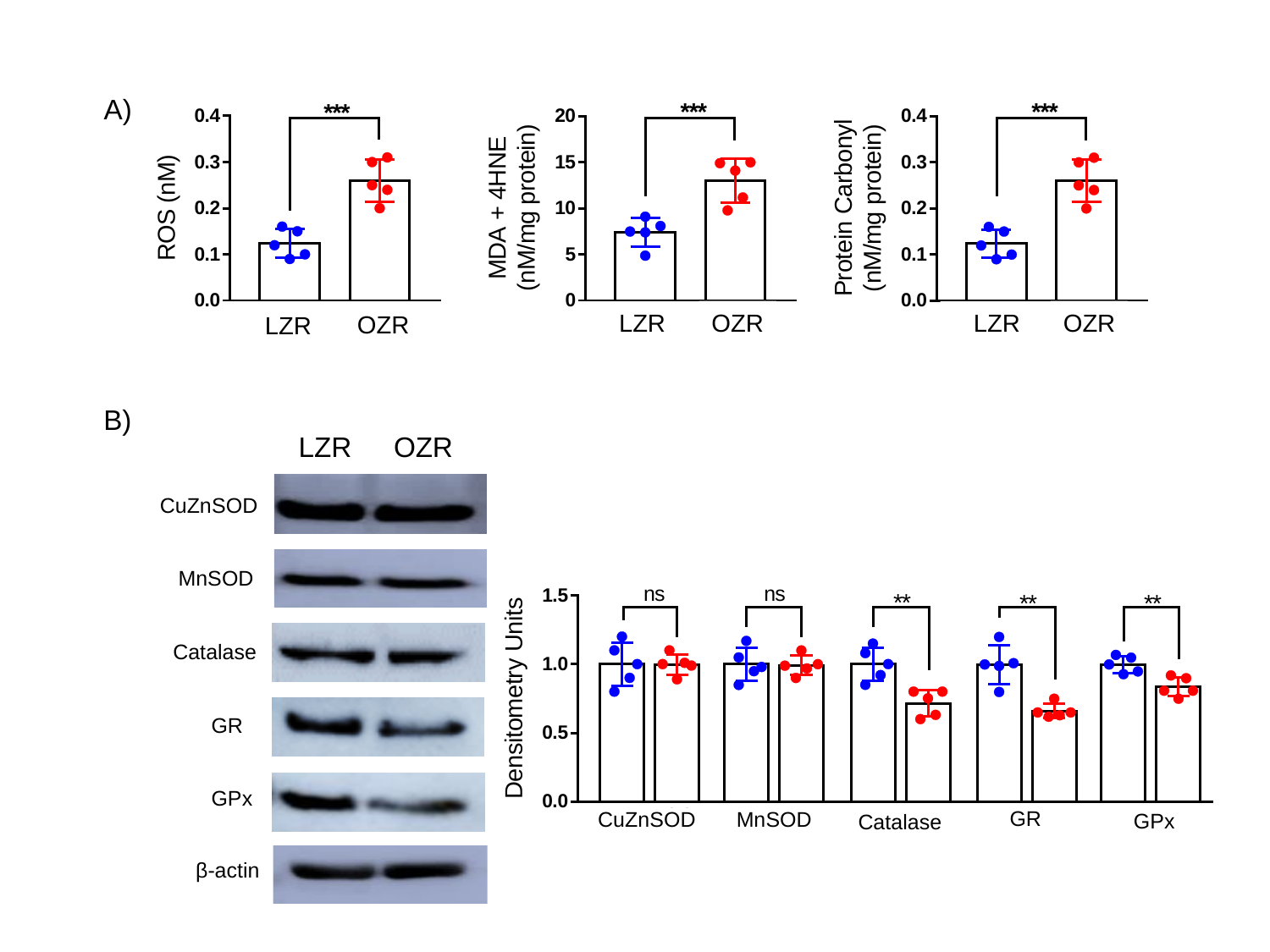

A)
OZR
OZR
LZR
LZR
OZR
LZR
B)
OZR
LZR
CuZnSOD
MnSOD
Catalase
GR
GPx
GR
CuZnSOD
MnSOD
GPx
Catalase
β-actin
