# Supplementary material for: Bladder Dysfunction in an Obese Zucker Rat: The Role of TRPA1 Channels, Oxidative Stress, and Hydrogen Sulfide
Source: Oxid Med Cell Longev. 2019 Aug 20;2019:5641645. doi: 10.1155/2019/5641645 (PMC6721245; doi:10.1155/2019/5641645)
Supplement: Supplementary 5 — Supplementary Figure 5: reduced CSE expression in the OZR bladder. Uncropped images of immunoblots of CSE and β-actin displayed in Figure 5(j) in the lean Zucker rat (LZR) and obese Zucker rat (OZR) (n = 6). The bands of interest are indicated by black boxes on the gels and show a reduced CSE expression in the OZR bladder. [file 5641645.f5.pptx]

## Slide 1
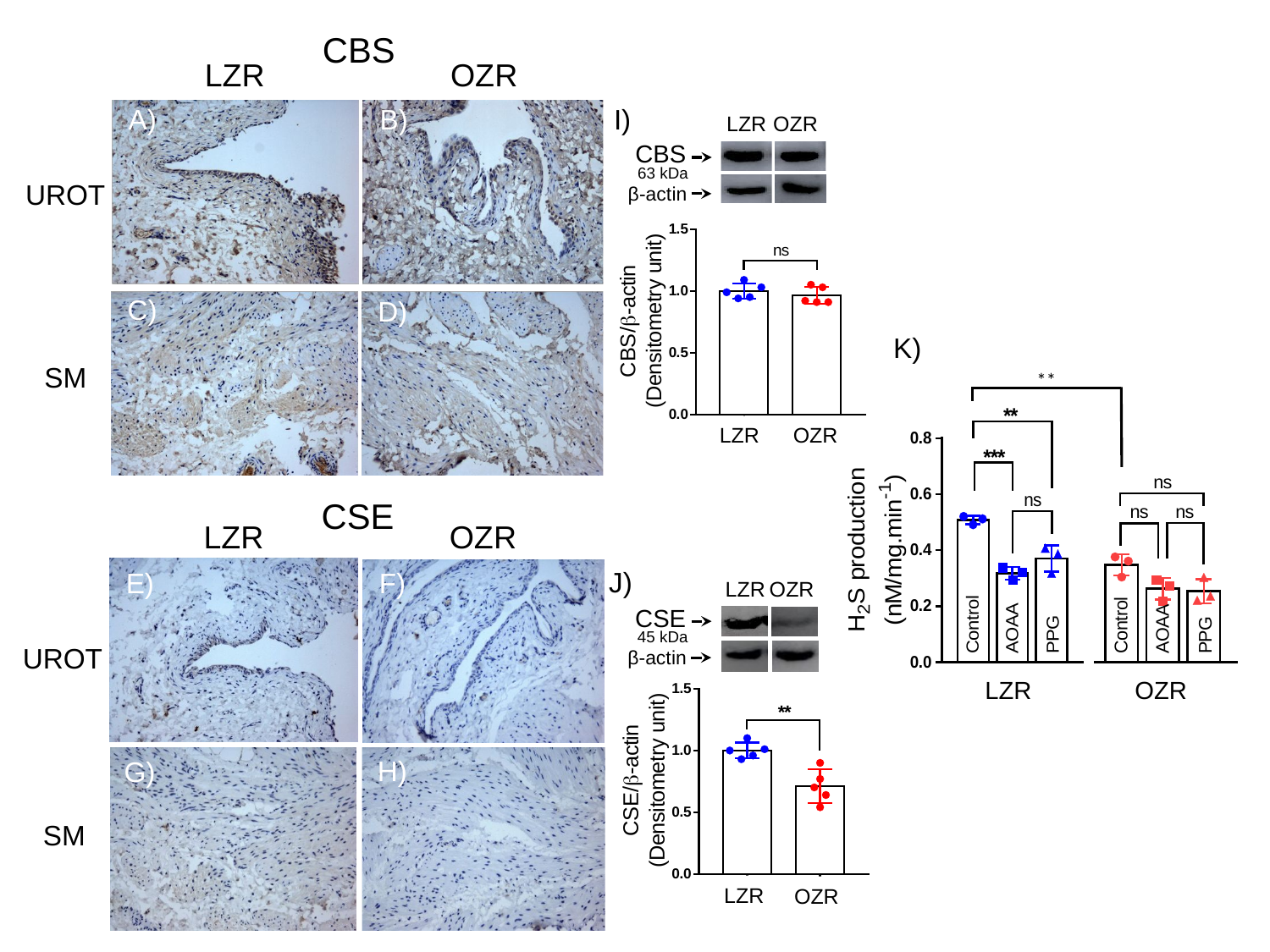

CBS
LZR
OZR
A)
B)
UROT
C)
D)
SM
I)
LZR
OZR
CBS
63 kDa
β-actin
K)
**
OZR
LZR
CSE
LZR
OZR
F)
E)
UROT
H)
G)
SM
J)
OZR
LZR
CSE
45 kDa
β-actin
LZR
OZR
LZR
OZR
